# Matrix regenerative therapy

**DOI:** 10.22336/rjo.2017.2

**Published:** 2017

**Authors:** Cristina-Mihaela Timaru, Cornel Stefan, Daniela Adriana Iliescu, Algerino De Simone, Mehdi Batras

**Affiliations:** * phthalmology Department, “Dr. Carol Davila” Central Military Emergency University Hospital, Bucharest, Romania

**Keywords:** regenerating agents, RGTA, extracellular matrix, wound healing

## Abstract

The extracellular matrix (ECM) is responsible for many of the cell behavior processes, including cell proliferation and growth, survival, change in cell shape, migration, and differentiation.

The most important component of the ECM is heparan sulfate (HS), because it insures the storage of many cell communication proteins, necessary for the continuous and identical renewal of cells and thus for tissue regeneration.

Regenerating agents (RGTA®) are bioengineered structural analogues of heparan sulfate glycosaminoglycans that replace the degraded endogenous HS of the ECM.

In the ophthalmological field, RGTA® represents an innovative approach for the improvement of the ocular surface wound healing and matrix remodeling and plays a role in controlling and regulating the wound healing process in various ocular diseases.

## Introduction

Corneal pathologies remain a major health concern in the ocular surface diseases. Improving both the quality and the speed of healing and controlling the inflammation are goals in treating the corneal injuries. 

The cornea is a complex structure responsible for three quarters of the optical power of the eye as well as one of the first lines of immunologic defense. The normal cornea is transparent, free of blood vessels, and densely innervated. It consists of the following layers:

1. **The epithelium:** stratified squamous and non-keratinized; the cells are interconnected by hemidesmosomes, critical in maintaining a physiological barrier.

2. **The Bowman layer:** acellular superficial layer formed by collagen fibers. 

3. **The stroma:** layers of collagen fibrils whose spacing is maintained by proteoglycan ground substance (glycosaminoglycans-GAGs: chondroitin sulfate, keratin sulfate, heparin sulfate) (the extracellular matrix); the regular arrangement and spacing is critical for optical clarity; 90% of corneal thickness; the mechanical response of the cornea to injury is dominated by the stroma; the stroma is approximately 78% water, 15% collagen and 7% non-collagenous proteins, proteoglycans and salts. 

4. **The Descemet membrane:** fine layer of collagen fibrils, different from the stromal collagen.

5. **The endothelium:** monolayer of polygonal cells that pump excess fluid out of the stroma. 

6. **The Dua layer:** a sixth layer between the stroma and the Descemet membrane; its existence was suggested by Harminder Singh Dua et al. in 2013, but many scientists are still conflicted [1,2].

The RGTA-based technology is a new therapeutic approach also named “Matrix Therapy”, aiming to preserve the extracellular matrix (ECM) and providing encouraging results in tissue regeneration.

ECM is an organized complex macromolecule network of proteins such as collagen and elastin that are linked together through polymers called glycosaminoglycans (GAGs).

The most important component of the ECM is heparan sulfate (HS). It insures the storage of many cell communication proteins such as growth factors and cytokines, necessary for the continuous and identical renewal of cells and thus for tissue regeneration. HS also provides a mechanical protection of the matrix signaling proteins against proteolytic degradation. 

## Corneal lesions healing and regenerating process

ECM is responsible for many of the cell behavior processes, including cell proliferation and growth, survival, change in cell shape, migration, and differentiation.

Corneal homeostasis (replacing a dead cell by a new identical cell) is regulated by the local cellular microenvironment. The initial endogenous signals needed for tissues to regenerate come from the matrix. They are expected to trigger the natural onset of events, signaling cells to migrate and multiply with the cascades and equilibrium found in tissue homeostasis to achieve a perfect replacement or regeneration.

Within the cornea, the ECM is continuously remodeled by cells via the degradation of the ECM components trough proteases, followed by the reassembly of the newly synthesized protein components secreted by cells. 

Corneal healing is a complex process involving cellular interaction and various molecules (proteases, growth factors, and epithelial and stromal cytokines).

When a lesion is produced, the GAGs are destroyed by the glycanases and can no longer be bound by the proteins, leaving them sensitive to proteases. The ECM architecture is disorganized and its function is disrupted. The inflammatory process determines inflammatory cells migration and release of less specific factors that replace growth factors. The homeostasis can no longer be effective and a healing process is starting [**3**].

The process of corneal epithelial wound healing can be divided into phases that occur in sequence, but may overlap in time:

1. ***Latent Phase:*** cellular remodeling and changes to tear composition in preparation for healing; there is an increased production of enzymes that decrease cellular adhesion and help enhance cellular migration; they are also important in the degradation and remodeling of normal extracellular matrix (ECM) maintenance [**4**,**5**].

2. ***Migration Phase.*** The next phase occurs as cells near the wound edge flatten and spread; contractile elements pull the cell forward toward the defect. Adjacent cells remain attached by desmosomes and maintain their position relative to each other as they slide across the denuded area. This cycle continues until the defect is completely sealed by a single layer of cells. The process typically takes place over 24 to 36 hours, though time can vary depending on the defect’s location and size [**6**]. 

3. ***Proliferation Phase.*** After migration is complete, the monolayer of cells covering the defect proliferates to restore the normal thickness and fill in the defect. Tight junctions form to re-establish the cornea’s barrier function, and gap junctions, adherens junctions and desmosomes reform between cells [5,6]. The reepithelization is initiated by the keratinocytes that proliferate, grow, and reform the tissue in depth. The deep tissue is reconstituted by proliferating fibroblasts that re-colonize the wound, synthesize a new temporary ECM, and form new blood vessels.

4. ***Remodelling Phase:*** can last for several years and consists of an ECM reorganization, vascular regression, and cell density reduction. Fibroblasts produce collagen as well as glycosaminoglycans and proteoglycans, which are major components of the ECM. One critical feature of the remodeling phase is ECM remodeling to an architecture that approaches that of the normal tissue [**7**].

Chronic and acute wounds heal very differently because of their physiology and repair characteristics.

Acute wound repair is a systematic process, consisting of homeostasis, inflammation, migration, proliferation and remodeling, while chronic wound repair is much more complex. Chronic wounds have a prolonged inflammatory phase, thus delaying the healing and repair process. In such wounds, an endless cycle of deterioration-healing-deterioration takes place. This precise action of the ECM is a potential target for RGTA® [**8**].

## ReGeneraTing Agents (RGTA)

***The RGTA concept***

Regenerating agents (RGTA®) are bioengineered structural analogues of heparan sulfate glycosaminoglycans (HS GAGs) that replace the degraded endogenous HS of the ECM. They are obtained by controlled grafting of carboxymethyl and sulfate groups on dextran polymers. Unlike naturally occurring HS, these polymers are stable and resistant to degradation [**9**]. 

They are adapted to interact with, and protect against proteolytic degradation of cellular signaling proteins (growth factors, cytokines, interleukins, colony stimulating factors, chemokines, and neurotrophic factors) and re-establish the intercellular links.

When a tissue is attacked, stressed cells release proteases and glycanases, which destroy this matrix architecture. Tissue-regenerating agents (RGTA) mimic the action of destroyed heparan-sulfate molecules, thereby recreating a matrix microenvironment in which cells can migrate and multiply. Moreover, these agents break the negative repair-destruction cycle occurring in chronic lesions [**10**].

The result is the preservation of the tissue natural endogenous signaling and is reflected by spectacular tissue regeneration or by a very greatly improved tissue repair.

These effects allow the creation of a suitable microenvironment for cells to respond properly to the cascade of signals needed for the tissue regeneration process to take place.

***Mechanism of action***

RGTA®s were designed to be a matrix therapy that restores the natural cellular microenvironment. They enhance both speed and quality of the tissue healing and lead in some cases to a tissue regenerating process.

The goal of this therapy is to block the cycle of ECM destruction and reconstruction that characterizes the chronic wounds by introducing a glycanases-resistant biopolymer engineered to mimic HS to improve tissue healing. This ECM stability is critical to the health and healing of wounds [**11**].

When applied topically to a wound, RGTA® penetrates into the micro-clefts of the damaged ECM, where it replaces the endogenous HS that have been degraded by glycanases. By binding to structural matrix proteins (collagen, elastin, fibronectin), the scaffolding properties of the ECM, and the mechanical protection of the matrix signaling proteins (heparin-binding growth factors, cytokines, neurotrophic factors) against proteolytic degradation, are restored [**12**]. This, in turn, prevents the degradation of the extracellular matrix proteins, and promotes stromal and, subsequently, epithelial healing [**13**,**14**]. 

## Regenerative medicine

There are multiple studies on a variety of animal species and tissue injury models including bone, gastrointestinal, muscular (including cardiac muscle), gingival, mouth and skin lesions, which proved that local or systemic administration of RGTA® improve the speed and quality of wound healing, in both epithelial and connective tissues. 

***RGTA®s in ophthalmology***

In the ophthalmological field, the available RGTA® is alfa 1-6 poly (carboxymethyl glucose sulfate) (**CACICOL**® – Laboratoires Thea). Several studies tested and proved the ability of RGTA® to promote the healing of chronic and severe corneal dystrophies, and healing of chronic corneal ulcers in humans. The heparan sulfate analog was shown to enhance re-epithelialization after corneal ulcer, reduce corneal inflammation and neovascularization, and suppress the antioxidant/ pro-oxidant imbalance in the injured corneal epithelium [**17**,**18**].

RGTA (Cacicol) is supplied as a sterile single-dose solution (0.33ml) of alpha 1-6 poly carboxymethyl glucose sulfate, with dextran T40 and sodium chloride as excipients. It contains no component of animal or biological origin, and penetrates into the cornea without crossing Descemet’s membrane. It was approved in the UE in 2008. 

It is distributed in an aluminum foil for protection against light, being stable for 36 months at a temperature between 4 and 25 degrees Celsius. 

***RGTA®s studies***

The first evidence of the efficacy of a RGTA® ophthalmic solution was obtained in a series of in vivo experiments on rabbit eyes that presented with alkali-induced severe corneal ulcers. A single drop of RGTA solution at a concentration of 100 µg/ mL was able to optimize the healing process, restoring an almost normal corneal histology after one week [**15**].

Since then, a high number of preclinical studies were performed by testing the efficacy of RGTA®s in different types of corneal lesions. 

Using the same lesion model, Takesue et al. (2005) revealed an improved healing with a decreased corneal opacity in mice after the topical application of RGTA® on corneal wound healing after burn injury [**19**].

A similar study was published by Cejkova J in 2014 on the effects of RGTA® therapy in rabbit corneas injured with alkali. The study demonstrated that RGTA® facilitates the healing of the alkali-injured corneas via a reduction of proteolytic, oxidative and nitrosative damage. The corneal thickness increased after the alkali injury and decreased during the corneal healing after RGTA treatment faster than after the placebo application. Following the injury with the high alkali concentration, corneal inflammation and neovascularization were highly pronounced in placebo-treated corneas, whereas in RGTA-treated corneas they were significantly suppressed. In conclusion, RGTA facilitates the healing of injured corneas via a reduction of proteolytic, oxidative and nitrosative damage [**16**].

Aifa et al. published a single-center, uncontrolled, prospective study in 2012 concerning the efficacy of RGTA therapy in corneal neurotrophic ulcers in 11 patients. The defect in 72.7% (8 patients) was completely healed in 8.7 weeks, the area of the lesion diminishing with 50% or more in the first week. No local or systemic side effects were noticed and the treatment was very well tolerated [**11**]. 

## Indications in ophthalmology 

This heparan mimetic, which stimulates extracellular matrix healing, may be a possible alternative therapy to heavy and invasive treatments such as autologous serum or amniotic membrane transplantation in patients suffering from:

- chronic corneal wound healing

- persistent and recurrent epithelial chronic defects

- corneal ulcers

- corneal dystrophies

- chemical burns

- traumatic injuries

- chronic wearing of contact lenses

- hereditary factors: familial dysautonomia, Riley-Day syndrome (loss of sensitivity, loss of tear secretion)

- limbus stem cells destruction

- viral keratitis

- sever dry eye syndrome

- old age (reduces corneal sensitivity, modifies the tear film, reduces the autonomous nervous system response)

- corneal surgery: refractive surgery, corneal transplant, cataract surgery

- toxic iatrogenic keratitis: topical anesthetics, preservatives, chronic medication (for glaucoma)

- autoimmune diseases (Sjogren syndrome, Lyell syndrome)

- corneal lesions in diabetes, malnutrition, alcoholism

- corneal ulcer in neovascular glaucoma.

## Administration

The number of heparan-binding sites available in wound tissue is limited, and once all these sites are occupied by RGTA, excess RGTA may compete with heparan-binding growth factors for sites on the matrix-bound RGTA. Thus, heparan-bound growth factors/ cytokines stored in the matrix could be removed from the matrix by this excess, hence reducing the amount of the growth factor available, and healing efficacy. For this reason, daily, or more than daily, addition does not seem to be needed [**11**].

The dosage depends on the etiology of the lesion. It can vary between:

- 1 drop/ week, for 1-2 months

- 1 drop/ 2 days, for 10 days

- 1 drop/ 2-3 days for 2-3 months or 1 drop/ day for a month in very difficult cases.

It has to be administered 15 minutes after another topical treatment, in the absence of an infectious disease and not associated with topical aminoglycosides (neomycin, gentamicin). 

## Our experience

26 patients with corneal lesions of various etiologies (infectious, posttraumatic, severe dry eye syndrome, post transplant, viral, linear corneal dystrophy, and post refractive surgery) were treated with RGTAs, 1 drop/ week for 2-4 months.

Follow-ups concentrated on symptoms evolution (cloudy vision, foreign body sensation, pain, excessive tearing), visual acuity and quality of life improvement, slit-lamp examination of the anterior segment, with fluorescein staining of the defect and defect measurements at each visit (maximum diameter) [**20**].

**Fig. 1:**
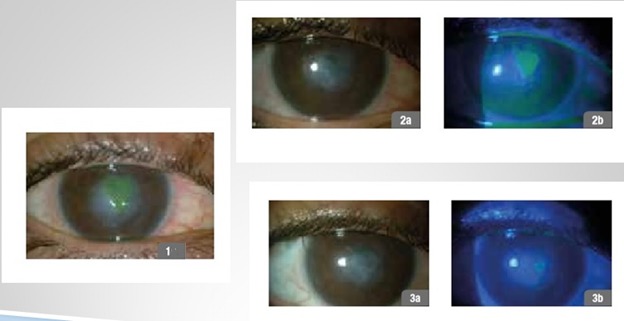
47-year-old female patient, infectious corneal ulcer; 1 - day 1 of treatment, 2a,b - follow-up day 30, 3a,b - follow-up day 60

**Fig. 2:**
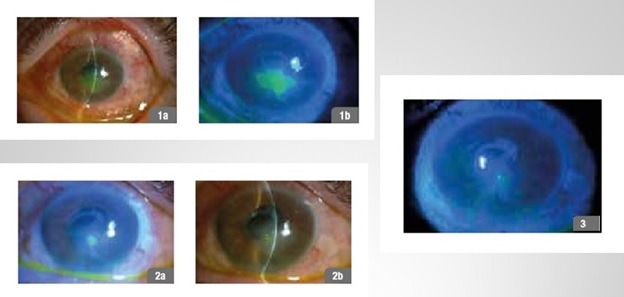
42-year-old male patient, linear corneal dystrophy; 1a,b - day 1 of treatment, 2a,b - follow-up day 30, 3 - follow-up day 90

**Fig. 3:**
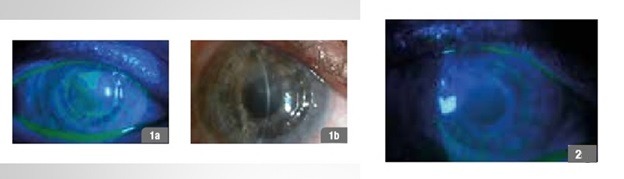
57-year-old male patient, post corneal transplant; 1a,b - day 1 of treatment, 2 - follow-up 60 days

**Fig. 4:**
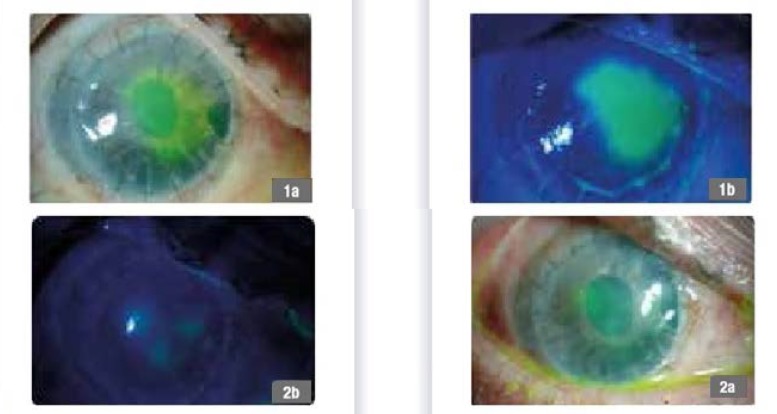
59-year-old male patient, post corneal transplant, 1a,b - day 1 of treatment, 2a,b - follow-up 90 days

**Fig. 5:**
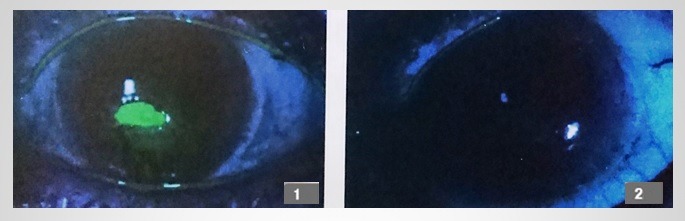
39-year-old female patient, post refractive surgery, 1 - day 1 of treatment, 2 -follow-up 30 days

**Fig. 6:**
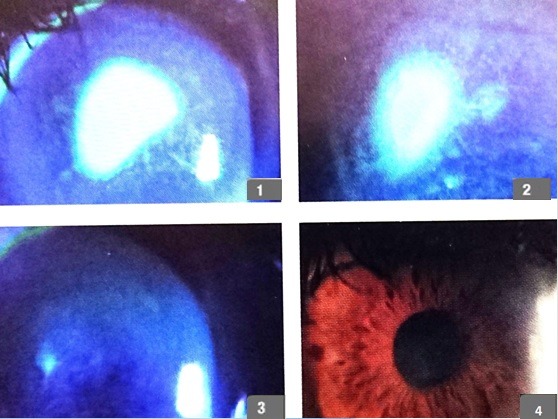
45-year-old female, severe dry eye syndrome, 1 - day 1 of treatment, 2 - follow-up 30 days, 3 - follow up 90 days, 4 - follow-up 120 days

**Fig. 7:**
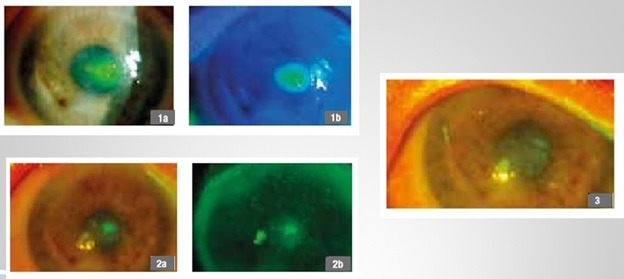
40-year-old female patient, viral keratitis, 1a,b - first day of treatment, 2a,b - follow-up at 30 days, 3 - follow-up at 90 days

## Results

The results are very promising. 

All the patients confirmed an improvement or even disappearance of symptoms and a better quality of life due to that effect. 

Objectively, visual acuity improved in all cases and the defect was cured in 73% (19 patients) of the cases and reduced by 50% or more in 27% (7 patients).

The easy way of administering it and the weekly dosage insured a good adherence to treatment of all patients. No side effects, local or systemic, were noticed and the therapy was well tolerated by patients [**21**].

## Conclusions 

In the ophthalmological field, RGTA® represents an innovative approach for the improvement of the ocular surface wound healing and matrix remodeling and plays a role in controlling and regulating the wound healing process in various ocular diseases, such as those involving corneal epitheliopathy, chemical or physical trauma, severe dry eye syndrome, scarring conjunctivitis, or after refractory surgery.

It is effective in improving subjective and objective symptoms, corneal healing and the patient’s comfort, has no noticed side effects and it is well tolerated by the patients. This therapy enhances both speed and quality of tissue healing, promoting tissue regeneration.
